# Reverse social contagion as a mechanism for regulating mass behaviors in highly integrated social systems

**DOI:** 10.1093/pnasnexus/pgae246

**Published:** 2024-06-26

**Authors:** Maurizio Porfiri, Pietro De Lellis, Eighdi Aung, Santiago Meneses, Nicole Abaid, Jane S Waters, Simon Garnier

**Affiliations:** Center for Urban Science and Progress, New York University, Brooklyn, NY 11201, USA; Department of Mechanical and Aerospace Engineering, Tandon School of Engineering, New York University, Brooklyn, NY 11201, USA; Department of Biomedical Engineering, Tandon School of Engineering, New York University, Brooklyn, NY 11201, USA; Department of Electrical Engineering and Information Technology, University of Naples Federico II, Naples 80125, Italy; Department of Biomedical Engineering and Mechanics, Virginia Tech, Blacksburg, VA 24061, USA; Department of Biological Sciences, New Jersey Institute of Technology, Newark, NJ 07102, USA; Department of Mathematics, Virginia Tech, Blacksburg, VA 24061, USA; Department of Biology, Providence College, Providence, RI 02918, USA; Department of Biological Sciences, New Jersey Institute of Technology, Newark, NJ 07102, USA

**Keywords:** activity regulation, metabolism, networks, scaling theory, social insects

## Abstract

Mass behavior is the rapid adoption of similar conduct by all group members, with potentially catastrophic outcomes such as mass panic. Yet, these negative consequences are rare in integrated social systems such as social insect colonies, thanks to mechanisms of social regulation. Here, we test the hypothesis that behavioral deactivation between active individuals is a powerful social regulator that reduces energetic spending in groups. Borrowing from scaling theories for human settlements and using behavioral data on harvester ants, we derive ties between the hypermetric scaling of the interaction network and the hypometric scaling of activity levels, both relative to the colony size. We use elements of economics theory and metabolic measurements collected with the behavioral data to link activity and metabolic scalings with group size. Our results support the idea that metabolic scaling across social systems is the product of different balances between their social regulation mechanisms.

**Significance Statement**

All social systems, from bacterial colonies to human societies, experience a phenomenon called mass behavior, which is the rapid adoption of similar conduct by all group members through social contagion. Mass behaviors can have catastrophic outcomes such as mass panic and stampedes. However, not all social systems are equally susceptible to mass behavior. Social insect colonies, whose extreme level of social integration has earned them the name of superorganisms, are even remarkably resistant to them. Our manuscript explains this high degree of stability in the context of activity regulation and suggests a link between patterns of social interaction in an ant colony and its ability to efficiently scale its total metabolic activity as its size increases.

## Introduction

*Social contagion*—the spread of behaviors throughout a group by means of neighbor-to-neighbor interactions (1)—is a powerful organizing principle in animal societies (2, 3). It results from processes of social imitation and/or social pressure that create a positive relationship between the number of neighbors performing a given behavior around a given individual and the probability that this individual starts engaging in that behavior. When repeated, social contagion can lead to the emergence of *mass behavior*, during which individuals rapidly adopt very similar conduct, sometimes over large spatial scales (4). These mechanisms are ubiquitous in social systems (2, 5, 6), such as human crowds clapping as one (7), ants excavating complex nest structures (8), and the aerial and underwater ballets of flocks of birds (9) and schools of fish (10).

**Fig. 1.**
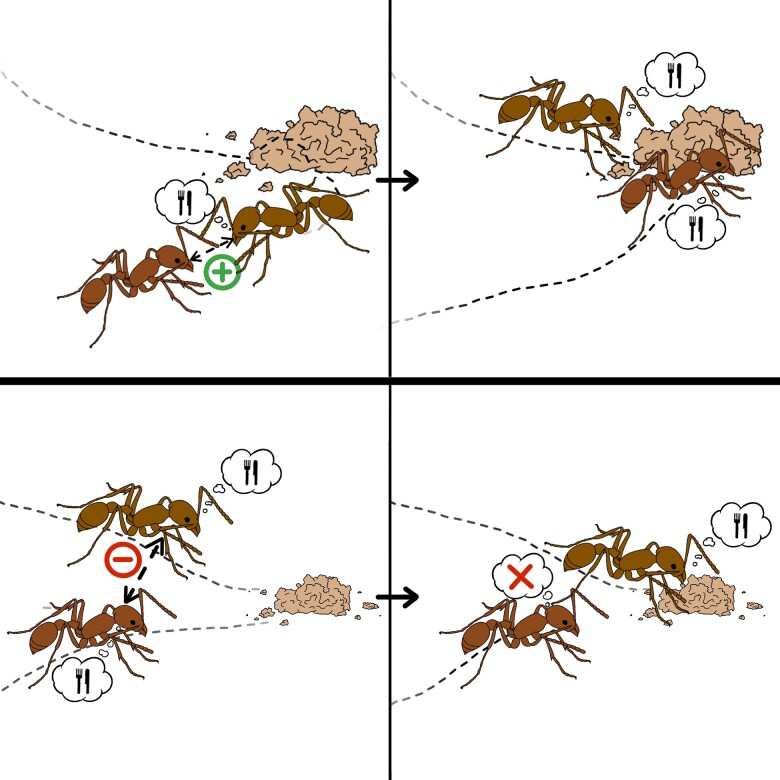
Illustration of the concepts of social contagion (top) and reverse social contagion (bottom). (top) An inactive ant interacts with an ant engaged in a foraging task: through social contagion (for example, caused by active recruitment), it also begins foraging. (bottom) Two ants engaged in foraging interact: through reverse social contagion (for example, caused by steric exclusion), one of them ceases their activity to become inactive. *Image credit: Isabella Muratore.*

Mass behaviors are prone to nonlinearities because of the amplifying effect of social contagion. Therefore, in highly integrated social systems (for example, social insect colonies, cooperatively breeding groups, and human societies), social contagion is often regulated to prevent the whole population from dedicating all its energy to a single behavior, especially if it has detrimental consequences (for example, stampedes (11, 12), futile cycles (13), and mass panic (14, 15)). One effective social regulation mechanism, often observed in the form of stop-signaling mechanisms (16) and blocking interactions (17), is achieved through behavioral deactivation between active individuals. Specifically, an individual engaged in a given behavior becomes more likely to interrupt its activity as it interacts with more neighbors engaged in the same behavior (18). We call this mechanism *reverse social contagion* (Fig. 1).

Here, we explore the role of reverse social contagion on activity regulation in colonies of harvester ants (*Pogonomyrmex californicus*). Using tracked video recordings of several colonies by Waters *et al.* (19), we observe that, on average, ants do not increase their level of individual activity with the size of the colony, despite an increasing number of social interactions at the colony level. To shed light on the mechanisms underlying this observation, we apply scaling theories commonly used for the study of human settlements (20–22) to the collected data. We derive scaling relationships that link the size of a colony to the organization of its network of interactions and activity levels, under the hypothesis that individual ants are subject to reverse social contagion. By leveraging this scaling, we show a potential link between activity and metabolism using flow-through respirometry data collected at the same time as the behavioral data. We conclude the paper with a parallel between an insect colony and a city, where social interactions lead to opposite allometric scalings for energy expenditure: hypometric (insect colonies) versus hypermetric (cities) scaling.

## Experimental materials and methods

**Fig. 2.**
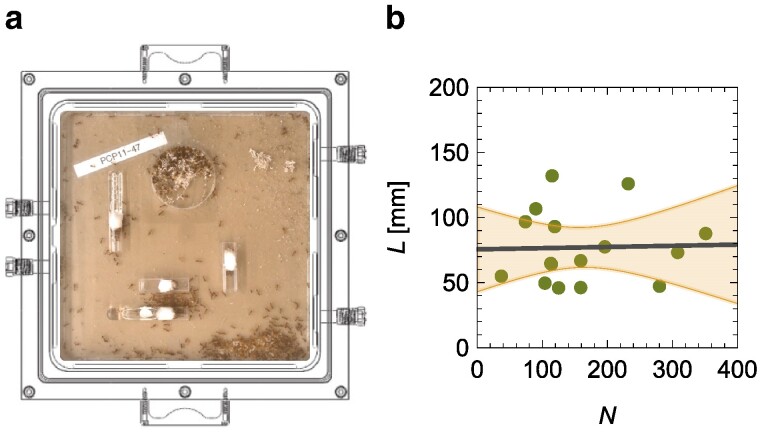
a) Experimental setup of Waters *et al.* (19) to study the metabolic rate of *Pogonomyrmex californicus* colonies as a function of individual ant movement, by placing the entire artificial nest enclosures where the colonies were reared into an airtight chamber with a clear lid, for simultaneous behavioral observation and flow-through respirometry measurements. b) Independence of the average length traveled by a worker, *L*, from the number of workers, *N*; the solid line is the linear fit and the shaded region is the 95% confidence interval.

We analyze experimental data of harvester ant colonies (*Pogonomyrmex californicus*) by Waters *et al.* (19) and partially re-analyzed by Toth *et al.* (23), combining simultaneous measurements of whole-colony metabolic rates via flow-through respirometry and video recordings of ants within their artificial nest enclosures (Fig. 2a). The video recordings make it possible to study the activity of and interactions among workers, while flow-through respirometry allows measurement of metabolic rates.

### Activity, interactions, and metabolism measurements

Twelve same-aged colonies were studied by Waters *et al.* (19), with sizes naturally ranging from a few dozen up to hundreds of workers. All the ant colonies started from queens of the California seed harvester ant. Video observations and flow-through respirometry were conducted in a lab maintained at 30–32° C. After each respirometry run, the wet mass of the colony was also determined.

To explore the causal mechanisms underlying allometric scaling of metabolic rate, these authors also tested size-reduced versions of the 12 colonies, obtained by removing 50% of the workers, larvae, and pupae, totaling 24 measurements over 16–24 hours of the metabolic rate as a function of the colony mass. The intervention of removing half of the colony retained all the members required for the colony to function while allowing for exploring the role of colony size. Waters *et al.* (19) showed that colony identity (paired before and after size-reduction) did not have a significant effect on metabolic rate, meaning that the data from colonies and their size-reduced versions are independent, and combining them does not run the risk of pseudoreplication.

For these 24 colonies, Waters *et al.* (19) acquired 30-second videos of 16 of them (eight whole and eight size-reduced colonies). Video observations were conducted in 248×248mm colony nest enclosures (one 30-second uncompressed AVI video per colony at a spatial resolution of 1,224×1,224 pixels and time resolution of 15 frames per second). These videos were manually tracked to score locomotory patterns of workers, and some of them were later examined by Toth *et al.* (23) to investigate interactions among workers. Here, we examine the whole set of 16 videos, refining and verifying those studied by Toth *et al.* (23) and completing the analysis of the remaining ones. In particular, for each video, we manually recorded the positions of each visually trackable worker (85% of the total number of counted workers (19), averaged over the colonies) to preserve their identity. The positions of individual workers were tracked using the plugin “MTrackJ” (from imagescience.org) from the imaging processing software “Fiji” (https://fiji.sc). Individuals received an identifying virtual tag on their mesosoma (alitrunk) every 5 frames in a stack of 450 frames (30-second videos at 15 frames per second). No physical identifiers such as paint or QR codes were used.

### Statistical analysis

Upon completing the video tracking for each colony, we ultimately had access to the metabolic rate (*B*), the mass (*M*), and individual trajectories for all tracked ants. The total number of workers ($N^{*}$) varied across colonies from 40 to 400. Of these workers, *N* were visually trackable, allowing for an estimation of the average path length traveled in the enclosure (*L*), the number of active individuals (*A*), the number of interactions between them based on spatial proximity (*E*), and the overall spanned area (Area). A worker was considered to be active if it moved at an average speed higher than $0.1\;\text{mm}\;{\text{s}}^{-1}$ during the video, corresponding to a traveled distance of more than 3mm over the 30 seconds. Spatial proximity was defined as being within a distance of d=6mm (approximately one body length).

For each frame, we created an undirected, unweighted network where two workers are connected if they are within the set distance and computed the total number of edges in the network. The number of interactions (*E*) was computed as the time average of the number of edges over the video. The area spanned by the workers was computed by dividing the enclosure into a 24×24 grid of square cells, each of length 10.6mm matching the area of interaction used to define the proximity network ($10.6^{2}≃\pi 6^{2}$). A robustness analysis for different interaction values for *d* and grid cell size is in the Supplementary material. To ensure the robustness of our claims with respect to the definition of interaction, we also considered antennal contacts (see Supplementary material). The complete dataset is included in Table S1 of the supplementary material.

Statistical analysis was performed using the built-in function “LinearModelFit” in “Mathematica,” yielding best-fit parameters (intercept and slope) with their 95% confidence intervals, coefficient of determination, and *p*-values for a *t*-distribution with 14 degrees of freedom (computed based on the number of colonies, 16, and the number of fitted parameters, two). Linear fits were computed on raw variables and the coefficient of determination was computed accordingly. Scaling laws, instead, were estimated on log-transformed variables, and the coefficients of determination was computed accordingly on log-transformed residuals. Coefficients of determination with respect to the comparison between experimental findings and theoretical predictions were calculated using raw or log-transformed variables, consistent with the corresponding figure illustrating the comparison.

## Results and discussion

### Activity regulation as a function of colony size

**Fig. 3.**
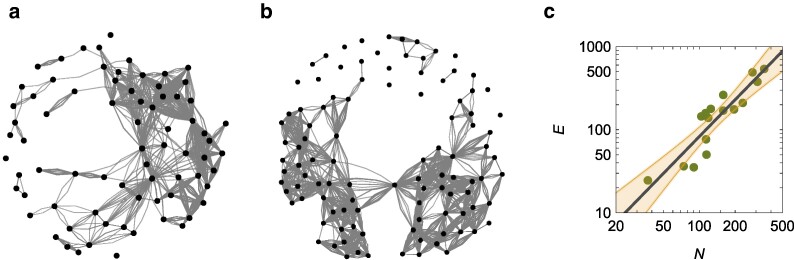
a) Representation of the spatial proximity network for the colony with 74 workers, showing an average degree of 0.982. b) Representation of the spatial proximity network for the colony with 104 workers, showing an average degree of 2.784. In a) and b), each node is a worker and the number of links between each pair identifies the number of frames in which they were within the chosen interaction distance. c) Hypermetric scaling of the number of interactions, *E*, versus the number of workers, *N*, for spatial proximity network; the solid line is the scaling $E\propto N^{1.47}$—in agreement with Rocha *et al.* (24)—and the shaded region is the 95% confidence interval.

The average path length does not vary with the colony size (Fig. 2b) [linear regression of *L* versus *N*: $R^{2}<0.01$ and p=0.915 for the slope]; an equivalent claim holds for the median length (see Supplementary material). This finding may be due to the average number of interactions experienced by a worker being independent of the colony size. As such, increasing the colony size would not alter the overall strength of the social contagion, whereby a worker would not experience a change in its number of interactions. However, this is unlikely the case based on the harvester ant literature, which shows ants exhibit inhomogeneous interaction patterns in a colony (25). We illustrate inohomogeneous interaction patterns in Fig. 3a and b, where we present interaction networks for two colonies with a different number of tracked workers (74 versus 104). For N=74, the average network degree (⟨k⟩=2E/N) is 0.982 while for N=104, it is 2.784.

By examining the entire dataset of 16 colonies, we observe hypermetric scaling of the number of interactions with respect to the colony size (Fig. 3c) [scaling of *E* versus *N*, $E=E_{0}N^{\beta_{E}}$: $R^{2}=0.81$, $\beta_{E}=1.47\pm 0.40$, and $E_{0}=0.0944$]. The observed scaling is in agreement with (24), who documented a universal scaling exponent of 3/2 for spatial proximity networks across animal taxa. Similar results are obtained with antennal contacts (see Supplementary material).

**Fig. 4.**
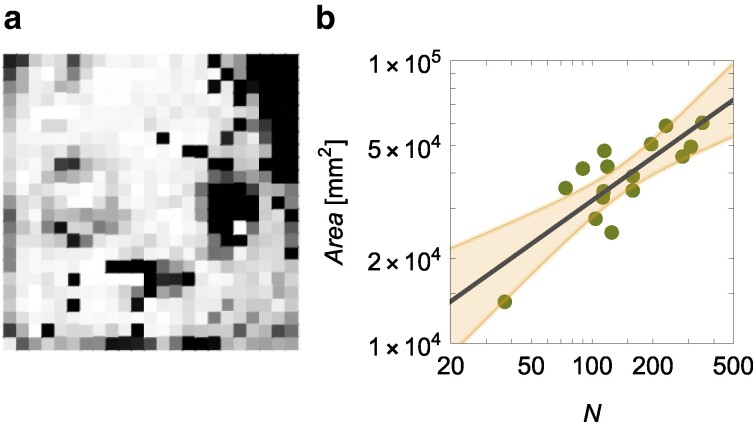
a) Colormap in gray scale of the spatial occupancy of workers in the next enclosure for the colony with 351 workers (white means locations never occupied by any worker and black location occupied the most). b) Hypometry of the area spanned by the workers, Area, versus the number of workers, *N*; the solid line is the scaling $Area\propto N^{0.51}$ and the shaded region is the 95% confidence interval.

The hypermetric scaling of the number of interactions is likely due to factors promoting the congregation of the ants, such as the formation of pheromone trails in the colony that do not uniformly cover the enclosure (26). As shown in Fig. 4a for the colony with N=351, workers do not uniformly disperse over the whole enclosure, rather, they spatially aggregate around a few locations (such as the corners of the enclosure, the brood pile, and test tubes with water and food). The area spanned by the workers scales hypometrically with the colony size (Fig. 4b) [scaling of Area versus *N*, $Area={Area}_{0}N^{\beta_{Area}}$: $R^{2}=0.65$, $\beta_{Area}=0.51\pm 0.21$, and ${Area}_{0}=3,050\;{\text{mm}}^{2}$]. Should ants distribute independently in the enclosure, $\beta_{Area}$ would approach zero, and, conversely, should ants tend to position themselves at a set, minimum distance with respect to each other, $\beta_{Area}$ would approach 1, as proposed by classical scaling theories (20). The observed scaling suggests an intermediate scenario.

For simplicity, assuming ants to be uniformly distributed in the spanned region, the overall number of interactions can be estimated as $\frac{1}{2}\frac{N^{2}a}{Area}$, where *a* is the effective interaction area of a worker and the factor of 1/2 avoids double-counting interactions. Thus, $\beta_{E}=1.47$ is explained by the scaling of the spanned area, namely, $\beta_{Area}≃2-\beta_{E}$. The effective interaction area should be $a=2{Area}_{0}E_{0}=576\;{\text{mm}}^{2}$, which is in between the values that one would predict by assuming either complete inactivity ($\pi d^{2}=110\;{\text{mm}}^{2}$, where *d* is the interaction distance) or steady motion ($2dL=924\;{\text{mm}}^{2}$, where *L* is the average path length in Fig. 2b).

Reverse social contagion is the process by which an active worker may cease their activity following an interaction with another active worker. Under the assumption of complete mixing, each worker interacts with ⟨k⟩=2E/N other workers that are active with probability A/N. The number of interactions of active workers with other active workers supporting reverse contagion is $\frac{\langle k\rangle A^{2}}{N}$. Once inactive, ants will become active again after a refractory period (27). We propose that reverse social contagion balances spontaneous activation, that is, $\frac{\langle k\rangle A^{2}}{N}=qN$, where *q* encapsulates the rates of spontaneous activation (positive relationship, the higher *q* the faster inactive ants spontaneously activate) and reverse social contagion (negative relationship, the higher *q* the more probable is that contact between two active ants results into one ceasing activity). It is tenable that the numerical values of model parameters, such as *q*, will depend on the ant species and even on the time of day of the experiments. For example, unlike *Pogonomyrmex* harvester ants in lab conditions (28), *Leptothorax* ants will activate together in rhythmic intervals of duration up to one hour. Calibrating our model on *Leptothorax* ants would thus require the selection of time-varying model parameters (27).

**Fig. 5.**
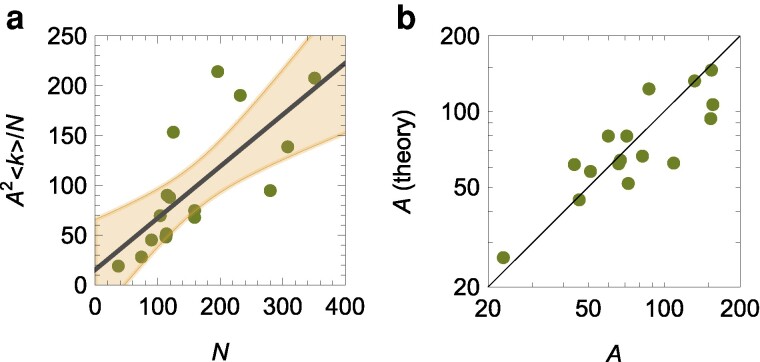
a) Evaluation of the hypothesis of reverse social contagion as a linear relationship between the number of interactions of active workers with other active workers, $\frac{\langle k\rangle A^{2}}{N}$, and the number of workers, *N*; the solid line is the linear fit and the shaded region the 95% confidence interval. b) Comparison between theoretical predictions and experimental observations of the number of active workers, *A*; the solid line is the bisectrix, indicating a perfect match.

We determine an association between the overall extent of the reverse social contagion and the colony size (Fig. 5a) [linear regression of $\frac{\langle k\rangle A^{2}}{N}$ versus *N*: $R^{2}=0.54$, p=0.526 for the intercept, and p=0.001 for the slope, q=0.519]. As a result of the hypermetric scaling of the number of interactions, we establish a hypometric scaling of the number of active ants in the form of


(1) $$A=\sqrt{\frac{q}{2E_{0}}}N^{\frac{3-\beta_{E}}{2}}≃1.66N^{0.77}.$$

Such a prediction is in agreement with experimental observations (Fig. 5b) [experimental data versus theoretical predictions: $R^{2}=0.71$], and it supports empirical and theoretical evidence that: (i) only a fraction of the workers are active and (ii) the disparity between active and inactive individuals increases with colony size (29, 30); equivalent predictions are obtained with antennal contacts (see Supplementary material). We note that the numerical values of *q* and $E_{0}$ bears no relevance on the hypometric scaling of *A* with respect to *N*, which is, in fact, ensured by $\beta_{E}$ being larger than 1.

### Linking activity regulation and metabolism

**Fig. 6.**
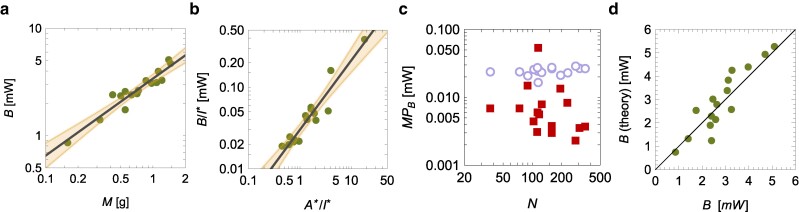
a) Hypometric scaling of metabolic rate, *B*, with colony mass, *M*; the black line is the scaling $B\propto M^{0.72}$, in agreement with Kleiber’s law for single organisms. b) Dependence of the metabolic rate per inactive workers, $\frac{B}{I^{*}}$, as a function of the ratio of active to inactive workers, $\frac{A^{*}}{I^{*}}$ (the numbers of active/inactive workers are scaled by the ratio between the total number of workers and the number of visible ones); the solid line is a Cobb–Douglas function of the form $B\propto (A^{*}{)}^{0.85}(I^{*}{)}^{0.15}$ and the shaded region is the 95% confidence interval. c) Marginal products of the number of active (open dots) and inactive workers (solid squares) on the overall metabolic rate, as predicted by the Cobb–Douglas function. d) Comparison between theoretical predictions and experimental observations of the total metabolic rate, *B*.

The scaling of the number of active workers is reminiscent of “Kleiber’s law” stating that $B\propto M^{3/4}$; such scaling is, in fact, observed in our experimental dataset (Fig. 6a) [scaling of *B* versus *M*: $R^{2}=0.90$ and $\beta_{B}=0.72\pm 0.13$]. We attempt to explore the relationship between Kleiber’s law and social reverse contagion by hypothesizing that the metabolic rate of the entire colony is controlled by the total number of active and inactive workers. By doing so, we neglect the queens, larvae, and pupae, which account for at most 20% of the total mass (19).

Energy expenditure of ants is known to increase with their speed, and running ants can spend as much as six times the energy than those at rest (31). As such, we opted to differentiate between the energy costs of active versus inactive workers in the prediction of the metabolic rate through a Cobb–Douglas function (32), where we treat the number of active workers as “labor input” for the colony (like the number of manual workers employed in American manufacturing for Douglas) and the number of inactive workers as the “capital input” of the colony (like the fixed capital in manufacturing for Douglas),


(2) $$B=B_{0}(A^{*}{)}^{\alpha_{B}}(I^{*}{)}^{1-\alpha_{B}}.$$

Here, we use scaled variables to acknowledge the fact that some of the ants that are not visible can contribute to the metabolic rate of the colony; namely, we introduce $A^{*}$ (the number of active workers multiplied by $N^{*}/N$) and $I^{*}$ (defined as $N^{*}-A^{*}$). The Cobb-Douglas function was fit using log-transformed variables (*B*, $A^{*}$, and $I^{*}$) as part of a univariate regression ($\ln\;\frac{B}{I^{*}}=\ln\;B_{0}+\alpha_{B}\ln\;\frac{A^{*}}{I^{*}}$), thereby controlling for multicollinearity between $A^{*}$ and $I^{*}$ (33) (we further verified the independence of their ratio on the number of workers, both *N* and $N^{*}$).

From the Cobb–Douglas function, we computed the marginal product of the number of active (inactive) workers on the overall metabolic rate, defined as the variation in metabolic rate due to one additional active (inactive) worker, while keeping the number of inactive (active) workers constant. In formulas, the marginal product of the number of active workers ($MP_{B}(A^{*})$) is


(3) $$MP_{B}(A^{*})=\frac{\partial B}{\partial A^{*}}=\alpha_{B}\frac{B}{A^{*}},$$

and the marginal product of the number of inactive workers ($MP_{B}(I^{*})$) is


(4) $$MP_{B}(I^{*})=\frac{\partial B}{\partial I^{*}}=(1-\alpha_{B})\frac{B}{I^{*}}.$$

A Cobb-Douglas function is compatible with the use of a scaling theory (21), facilitating the mathematical development of our approach and accurately describing the observed metabolic response (Fig. 6b) [scaling of $\frac{B}{I^{*}}$ versus $\frac{A^{*}}{I^{*}}$: $R^{2}=0.90$, $\alpha_{B}=0.85\pm 0.15$, and $B_{0}=0.0301\;\text{mW}$]. Predictably (31), active workers have a nearly three-fold effect of inactive ones, as seen from their marginal product on metabolic rate (Fig. 6c).

Based on the Cobb–Douglas function and the reverse social contagion, we expect that


(5) $$B=B_{0}N^{*}{\left(\sqrt{\frac{q}{2E_{0}}}\right)}^{\alpha_{B}}N^{\frac{(1-\beta_{E})\alpha_{B}}{2}}{\left(1-\sqrt{\frac{q}{2E_{0}}}N^{\frac{1-\beta_{E}}{2}}\right)}^{1-\alpha_{B}}.$$

Such a prediction is met by experimental observations (Fig. 6d) [experimental data versus theoretical predictions: $R^{2}=0.73$].

Assuming that $N^{*}/N\;\propto 1$ and M∝N, for N≫1, we establish $B\propto M^{\frac{(1-\beta_{E})\alpha_{B}}{2}+1}≃M^{0.80}$, in agreement with Kleiber’s law; equivalent predictions are obtained with antennal contacts (see Supplementary material). Within this framework, the hypometric scaling of metabolism relies on the network of interactions to scale hypermetrically, whereby we predict that B∝M if $\beta_{E}=1$. Perhaps, this is the case for groups of disconnected (34) or haphazardly collected (30) individuals, where the metabolic rate grows isometrically with the colony size.

### A parallel between social insect colonies and human urban centers

Urban settlement theories were the basis upon which we formulated our predictions. Like with metabolic scaling in insect colonies, most urban properties (*Y*) scale allometrically with a city’s population (*N*) as $Y\propto N^{\beta }$; for example, income scales hypermetrically with population (positive allometry). The simplest model to explain urban scaling is the amorphous settlement model, in which “the benefits of social interaction balance movement costs for a given population size” (22). Within this model, the cost of movement of the entire population (*C*) in a city of areal extension A is proportional to the city radius so that $C\propto NA^{1/2}$. The benefit of social interaction is Y∝E , where *E* is the total number of interactions, which scales as $N^{2}/A$. Equilibrium between *C* and *Y* yields $C=Y\propto N^{4/3}$ and $A\propto N^{2/3}$. Thus, the overall cost of transport for the population—a proxy for a city’s “metabolism”—grows hypermetrically with the population size with a scaling exponent of 4/3. For models of modern human settlements, the scaling of power dissipation (which includes moving people, goods, and information) remains hypermetric but the exponent reduces to 7/6 (20). This is in stark contrast with the hypometric metabolic scaling of insect colonies.

**Fig. 7.**
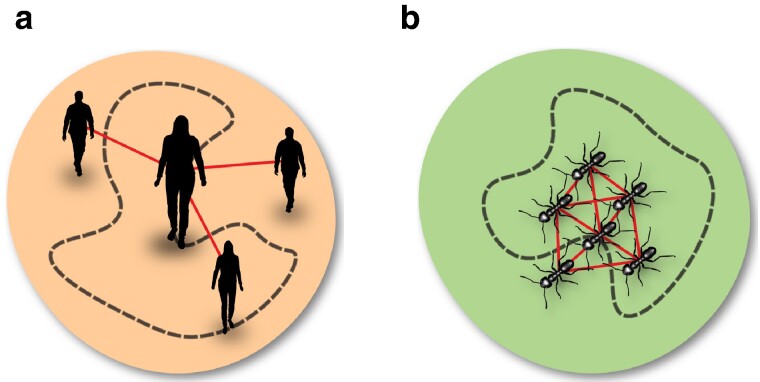
a) Illustration of an urban settlement composed of individuals who act for their own benefits: each person transforms their cost of movement (measured in some form of currency depending on their means of transport, C/N) into their own social interactions (proxied by the average connectivity, ⟨k⟩=2E/N). b) Illustration of a social colony of insects acting as a superorganism: each insect adjusts its energy expenditure, B/N, in response to its average connectivity so that it will increase its expenditure in response to reductions in connectivity. *Image credit: Anna Sawulska.*

A balance like the one underlying the amorphous settlement model cannot hold for the ant colonies: the metabolic rate *B* scales hypometrically with *N*, while the number of interactions *E* scales hypermetrically. One way to interpret the relationship between the total cost of movement and social benefits is by looking at the colony as one superorganism, rather than a collection of many, distinct organisms. Through this lens, the social benefit is the one of the collective, which seeks to maintain an average connectivity level in the colony: the higher the average connectivity, the lower the energy expenditure of a worker. While each human will want to transform their costs into their own interactions (Fig. 7a), insects may sacrifice their efforts for the benefit of the colony (Fig. 7b).

## Conclusions

Unlike in human settlements, the results of this study support the idea that ants act to control their energy spending at the level of the colony rather than the individual. This view is backed by the close matching between scaling laws derived from a model of reverse social contagion, which captures colony-level activity regulation, as well as the recovery of metabolic scaling with colony mass, in line with Kleiber’s law. Therefore, this work suggests that the appropriate atomic unit for an ant is its colony—and not itself as a single organism—and that social interactions between individuals and the overall colony metabolism are inextricably linked. Future work to further explore this relationship will include time-resolved models of social contagion and inhibition which capture the dynamics of these regulatory interactions and the energy savings they enable, as well as detailed observations to identify the proximal mechanisms underlying reverse social contagion in ant colonies.

## Supplementary Material

pgae246_Supplementary_Data

## Data Availability

Data needed to generate the results presented are included in supplementary material, in a compact form, and publicly available via Zenodo (https://zenodo.org/doi/10.5281/zenodo.11622717), in a more complete form. The code created to generate results is also available via Zenodo (https://zenodo.org/doi/10.5281/zenodo.11622717).
