# Supplementary material for: Reverse social contagion as a mechanism for regulating mass behaviors in highly integrated social systems
Source: PNAS Nexus. 2024 Jun 26;3(7):pgae246. doi: 10.1093/pnasnexus/pgae246 (PMC11220668; doi:10.1093/pnasnexus/pgae246)
Supplement: pgae246_Supplementary_Data [file pgae246_supplementary_data.docx]

**
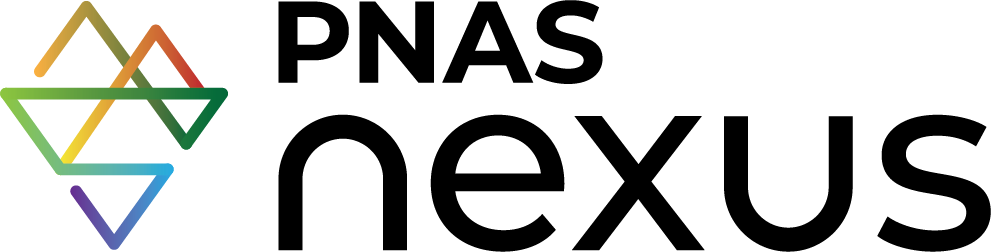
**

**Supplementary Information for**

Reverse social contagion as a mechanism for regulating mass behaviors in highly integrated social systems

Maurizio Porfiri, Pietro De Lellis, Eighdi Aung, Santiago Meneses, Nicole Abaid, Jane S. Waters and Simon Garnier

Corresponding author: Maurizio Porfiri

Email: [mporfiri@nyu.edu](mailto:mporfiri@nyu.edu)

**This PDF file includes:**

Supplementary text

Figures S1 to S2

Table S1

SI References

**Supplementary Information Text**

**Robustness analysis with respect to variations in the definition of proximity.** Varying the distance used to define spatial proximity does not affect our results on the hypermetric scaling of the number of interactions ($E$). Specifically, we varied the distance from $3$ to $12$ $\mathrm{mm}$ in steps of $1.5 \mathrm{mm}$ and registered a scaling exponent ($\beta_{E}$) varying in the relatively narrow range $[1.47,1.59]$, with the smallest value at $7.5 \mathrm{mm}$ and the largest at $12 \mathrm{mm}$. The coefficient of determination of the fit monotonically increases with the distance from $0.67$to $0.88$. In all cases, we recorded a scaling indistinguishable from a 3/2 scaling law. For the weakest fit (the smallest interaction distance), the 95% confidence interval on the scaling exponent was only marginally different from an isometry; for any other distance values, we recorded a hypermetry.

We performed an equivalent analysis for the area spanned by the workers. Specifically, we employed the same values of the distances to construct an array of grids for the enclosure, with each square cell having the same area as the interaction area around a worker. The finest resolution corresponded to a grid of cell length $5.3 \mathrm{mm}$ and the coarsest to $21.3 \mathrm{mm}$. For all the explored grids, we recorded hypometric scaling of the area spanned by the workers with scaling exponent ($\beta_{Area}$) monotonically decreasing from $0.69$ to $0.31$ as the grid became coarser. The coefficient of determination displayed an equivalent trend, decreasing from $0.70$ to $0.51$. In all cases, we recorded a hypometry, indistinguishable from a 1/2 scaling law for all grids except for the coarsest one.

These findings support that $\beta_{E}\cong3/2$ and $\beta_{E}+\beta_{Area}\cong2$, irrespective of the definition of the interaction distance.

**Verification of predictions using antennal contacts rather than spatial proximity.** We verified our predictions on hypermetric scaling of the number of active workers and total metabolic rate by examining antennal contacts, rather than physical proximity in the definition of an interaction. Specifically, antennal contacts (antennae of an ant touching other ants) were manually scored by playing videos backward and forward frame by frame and following the movements of each worker. For ten of the 16 colonies, antennal contacts between workers were already scored by Toth *et al*. [1]. In agreement with results in the main manuscript, we register hypermetric scaling of the number of interactions established by the workers with respect to the colony size (Fig. S1a) [scaling of $E$ versus $N$: $R^{2}=0.88$, $\beta_{E}=1.61\pm0.35$, and $E_{0}=0.0612$].

This alternative definition of interaction does not challenge the hypothesis of reverse social contagion (Fig. S1b) [linear regression of $\frac{{\left\langle k \right\rangle A}^{2}}{N}$ versus $N$: $R^{2}=0.32$, $p=0.776$ for the intercept, and $p=0.022$ for the slope, $q=0.844$]. Hence, we predict hypometric scaling of the number of active ants in the form of $A=2.63N^{0.70}$, in agreement with experimental observations (Fig. S1c) [$R^{2}=0.69$]. Likewise, implementing the Cobb-Douglas function to retrieve the metabolic rate from the number of active ants yields predictions in agreement with experimental observations (Fig. S1d) [experimental data versus theoretical predictions: $R^{2}=0.62$].

**Complete dataset.** All colony masses ($M$), metabolic rates ($B$), number of workers (measured, $N^{*}$, and tracked, $N$), interactions ($E$), and number of active ants ($A$) are reported in Table S1.

**Independence of median path length traveled from colony size.** The speed of workers is known to follow a non-normal distribution in the form of a power law [2]. Changing the mean to the median does not alter the claim in Fig. 2b of the main manuscript, whereby we confirm the independence of the median path length traveled by a worker ($L$) from the colony size ($N$) (Fig. S2) [linear regression of $L$ versus $N$: $R^{2}=0.07$ and $p=0.340$ for the slope].

**Fig. S1.**

(a)

(b)

**
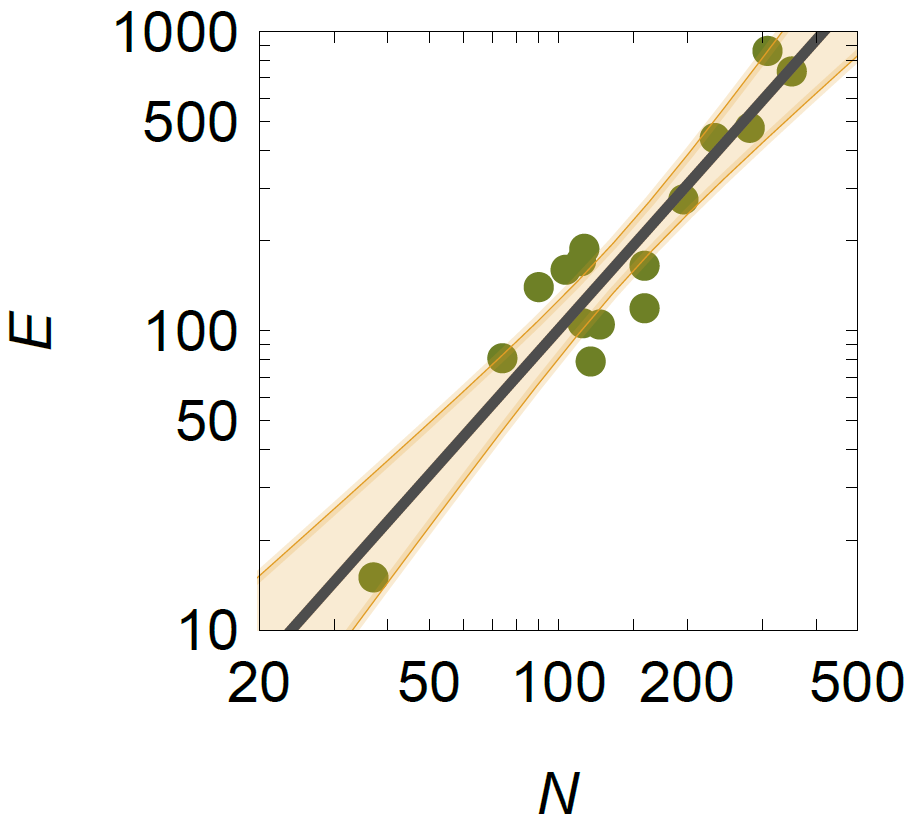

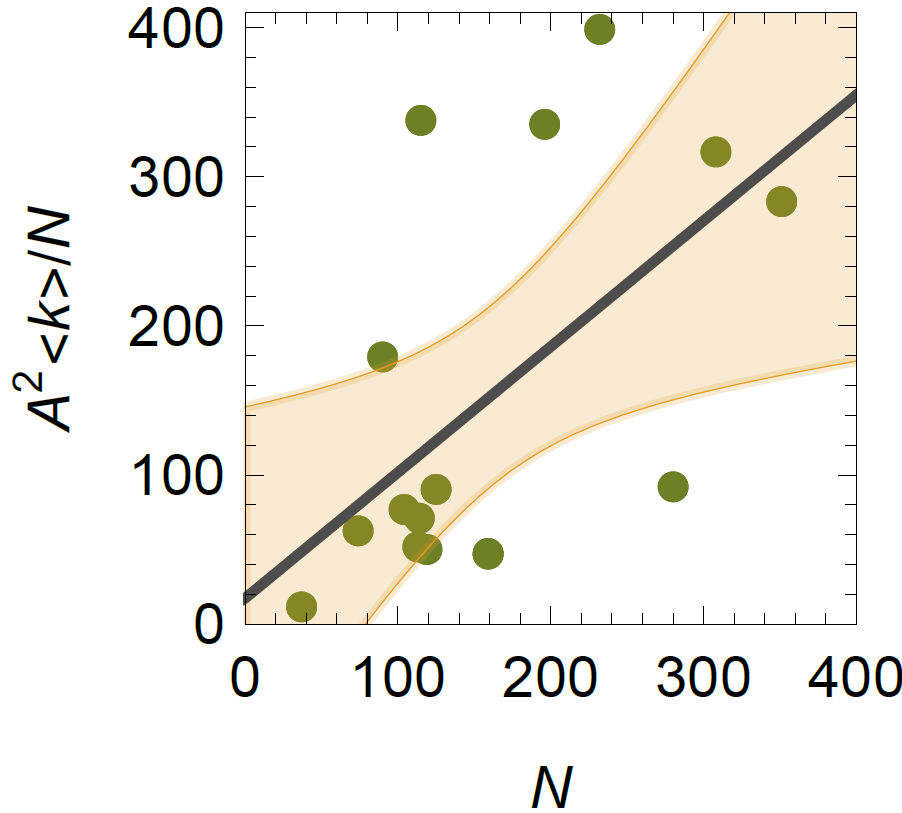
**

(d)

(c)

**
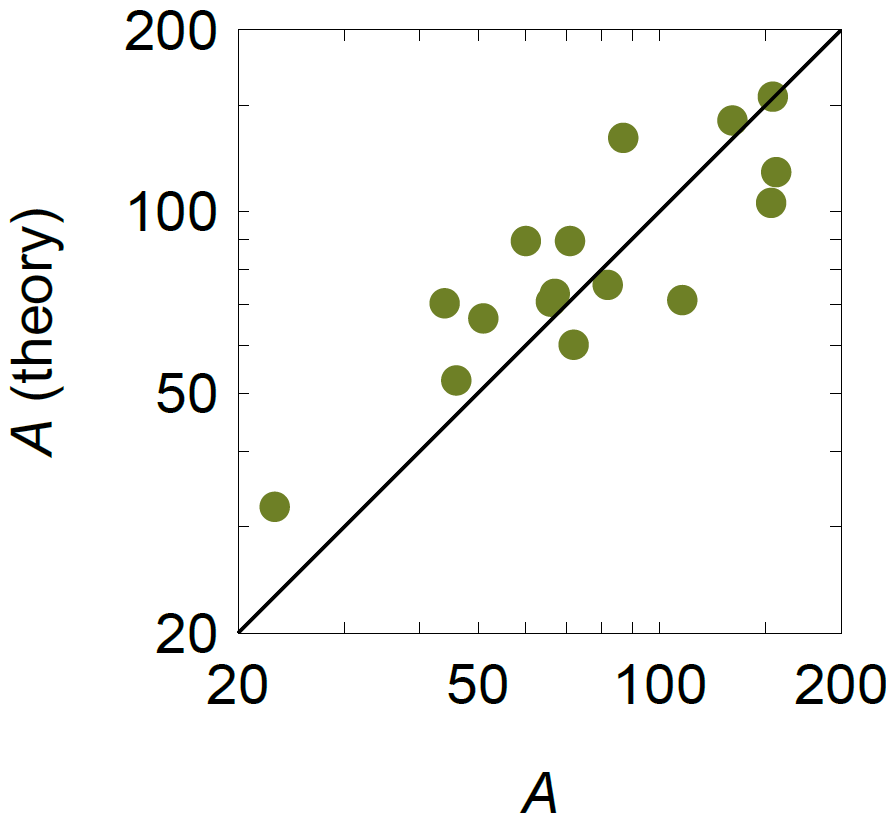

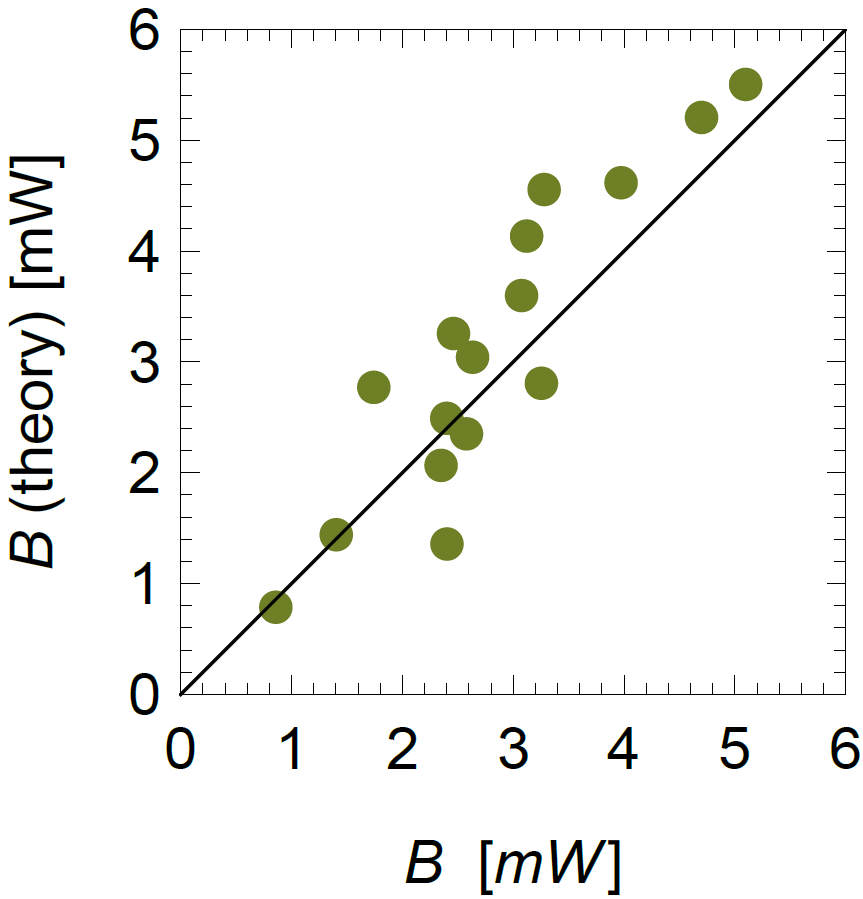
**

(a) Hypermetric scaling of the number of interactions, $E$, versus the number of workers, $N$, for antennal contacts; the solid line is the scaling $E\sim N^{1.61}$ and the shaded region is the 95% confidence interval. (b) Evaluation of the hypothesis of reverse social contagion as a linear relationship between the number of interactions of active workers with other active workers, $\frac{{\left\langle k \right\rangle A}^{2}}{N}$, and the number of workers, $N$, using antennal contact as a proxy of interaction; the solid line is the linear fit and the shaded region the 95% confidence interval. (c) Comparison between theoretical predictions and experimental observations of the number of active workers, $A$, using antennal contact as a proxy of interaction; the solid line is the bisectrix, indicating a perfect match. (d) Comparison between theoretical predictions and experimental observations of the total metabolic rate, $B$, using antennal contact as a proxy of interaction.

**Fig. S2.**

**
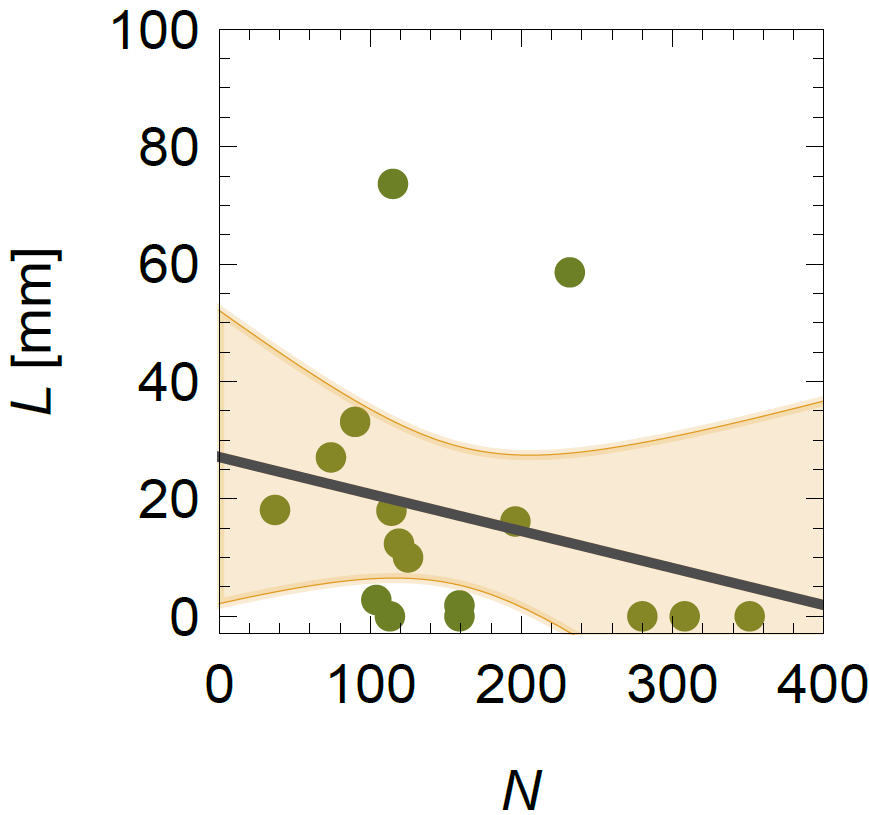
**

Independence of the median length traveled by a worker, $L$, from the number of workers, $N$; the solid line is the linear fit and the shaded region is the 95% confidence interval.

**Table S1.** Colony ID, mass ($M$), metabolic rate ($B$), number of visible workers tracked in the videos ($N$), total number of workers ($N^{*}$), mean and median path lengths traveled in the enclosure ($L$), number of interactions between them based on spatial proximity and antennal contact ($E$), area spanned by the workers ($Area$), and number of active workers ($A$). Note that traveled lengths, areas spanned, and interactions are computed for the ants that were visible during the videos.

| Colony ID | $M [g]$ | $B [\mathrm{mW}]$ | $N^{*}$ | $N$ | $L [\mathrm{mm}]$ (mean) | $L [\mathrm{mm}]$ (median) | $E$ (proximity) | $E$ (contact) | $Area [\mathrm{mm}^{2}]$ | $A$ |
| --- | --- | --- | --- | --- | --- | --- | --- | --- | --- | --- |
| pcp1105c | 0.97 | 3.07 | 239 | 232 | 126 | 59 | 210.5 | 441 | 58,800 | 156 |
| pcp1105m | 0.5 | 2.35 | 120 | 119 | 93.1 | 12.4 | 138.8 | 79 | 42,190 | 67 |
| pcp1107c | 1.12 | 3.97 | 326 | 308 | 73.2 | 0 | 377.5 | 862 | 49,540 | 132 |
| pcp1107m | 0.55 | 1.74 | 157 | 104 | 49.6 | 2.8 | 144.8 | 160 | 27,600 | 51 |
| pcp1114c | 0.86 | 3.25 | 162 | 115 | 132 | 73.7 | 50.2 | 188 | 47,950 | 109 |
| pcp1114m | 0.42 | 2.4 | 75 | 90 | 106.7 | 33.1 | 35.3 | 140 | 41,510 | 72 |
| pcp1121c | 1.46 | 4.7 | 360 | 280 | 47.3 | 0 | 490.8 | 477 | 45,800 | 87 |
| pcp1121m | 0.73 | 2.63 | 175 | 113 | 64.7 | 0 | 158.7 | 171 | 32,800 | 44 |
| pcp1123c | 0.32 | 1.4 | 83 | 114 | 64.1 | 18 | 76.7 | 106 | 34,490 | 66 |
| pcp1123m | 0.16 | 0.86 | 40 | 37 | 55 | 18.1 | 24.6 | 15 | 14,020 | 23 |
| pcp1143c | 1.08 | 3.12 | 254 | 159 | 46.2 | 1.86 | 170.1 | 119 | 34,720 | 71 |
| pcp1143m | 0.55 | 2.58 | 126 | 74 | 96.8 | 27.1 | 36.4 | 81 | 35,400 | 46 |
| pcp1147c | 1.4 | 5.1 | 400 | 351 | 87.7 | 0 | 539.3 | 736 | 60,280 | 154 |
| pcp1147m | 0.71 | 2.46 | 200 | 159 | 66.8 | 0 | 262.3 | 165 | 39,020 | 60 |
| pcp1154c | 1.21 | 3.28 | 292 | 196 | 77.5 | 16.1 | 176.7 | 275 | 50,670 | 153 |
| pcp1154m | 0.63 | 2.4 | 146 | 125 | 46 | 10 | 178.2 | 105 | 24,660 | 82 |

**SI References**

1. J.M. Toth, J. H. Fewell, J. S. Waters. Scaling of ant colony interaction networks. *Frontiers in Ecology and Evolution*, 10, 993627 (2023).
2. J. S. Waters, C. T. Holbrook, J. H. Fewell, J. F. Harrison. Allometric scaling of metabolism, growth, and activity in whole colonies of the seed-harvester ant *Pogonomyrmex californicus*. *The American Naturalist*, 176, 501–510 (2010).
